# Lesion-level dual-tracer PET biomarkers predict prognosis in multiple myeloma treated with CXCR4-directed radiopharmaceutical therapy

**DOI:** 10.1007/s00259-026-07814-5

**Published:** 2026-02-18

**Authors:** Song Xue, Sabrina Kraus, Johanna S. Enke, Kerstin Michalski, Marcus Hacker, Hermann Einsele, Andreas K. Buck, Constantin Lapa, Xiang Li, Niklas Dreher

**Affiliations:** 1 Division of Nuclear Medicine, Department of Biomedical Imaging and Image-guided Therapy, Vienna General Hospital, Medical University of Vienna, Vienna, Austria; 2 Department of Internal Medicine II, University Hospital Würzburg, Würzburg, Germany; 3 Nuclear Medicine, Faculty of Medicine, University of Augsburg, Augsburg, Germany; 4 Department of Nuclear Medicine, University Hospital Würzburg, Würzburg, Germany; 5 Department of Nuclear Medicine, Beijing Chest Hospital, Capital Medical University, Beijing, China

**Keywords:** Dual-tracer PET, CXCR4-RPT, Lesion-level quantification, Prognosis biomarker

## Abstract

**Background:**

C-X-C motif chemokine receptor 4 (CXCR4)-directed radiopharmaceutical therapy (RPT) represents a promising option for hematologic malignancies. Nevertheless, responses in relapsed and refractory (r/r) multiple myeloma (MM) are heterogenous, emphasizing the need for optimized patient selection before RPT. Current approaches mostly rely on qualitative assessments, confirming relevant CXCR4-expression by CXCR4-directed PET in vital myeloma burden, the latter usually being determined by additional [^18^F]FDG-PET.

**Purpose:**

To evaluate whether quantitative imaging biomarkers derived from dual-tracer PET/CT can enhance patient stratification and predict therapeutic response and survival following CXCR4-RPT.

**Materials and methods:**

22 patients with r/r MM who underwent CXCR4-directed [⁶⁸Ga]Ga-PentixaFor-PET/CT and [¹⁸F]FDG-PET/CT imaging prior to CXCR4-RPT were retrospectively analyzed. A fully automated pipeline performed deep-learning–based lesion segmentation and dual-tracer fusion; batch quality control and correction ensured segmentation accuracy and concordant-lesion adjudication (> 10% volumetric overlap) prior to lesion-level feature extraction. Features included demographics, laboratory/genetic variables, and imaging metrics from FDG- and CXCR4-PET, stratified by anatomic site (medullary vs. extramedullary) and concordance (concordant vs. discordant as defined by comparing FDG- and CXCR4-positive myeloma lesions). Surface-standardized maximum inter-lesion distances (sDmax) were additionally computed. Endpoints were therapy response (responder vs. non-responder as defined by serological response assessment based on IMWG-criteria or PET/MRI based response assessment) and overall survival (OS). Group comparisons were performed using Welch’s t-test/Chi-square; survival analysis was conducted applying Kaplan–Meier estimates and log-rank tests. Decision-tree models were interpreted with SHapley Additive exPlanations (SHAP).

**Results:**

Responders to RPT showed lower [^18^F]FDG-SUV_mean_ in medullary and extramedullary concordant lesions (Welch’s *p* = 0.03). In the response classifier, these metrics ranked among the top predictors by SHAP, alongside selected extramedullary CXCR4-dominant discordant features and high-risk cytogenetics. Shorter OS was associated with higher TLG[FDG medullary concordant], greater sDmax[FDG], and higher CXCR4-positive medullary burden (MTV/TLC) (log-rank *p* < 0.05). SHAP directionality agreed with univariate trends. BMI showed a modest inverse association with early mortality in the 6-month survival model.

**Conclusions:**

In our exploratory analysis, [¹⁸F]FDG-uptake within medullary concordant lesions was linked to both response and survival, while CXCR4-expressing medullary volume and sDmax added survival information. Concordance-aware, lesion-level quantification from dual-tracer PET/CT might present as a promising approach to aid risk stratification for CXCR4-directed RPT. However, initial findings of our hypothesis generating analysis warrant prospective validation in larger cohorts.

**Graphical abstract:**

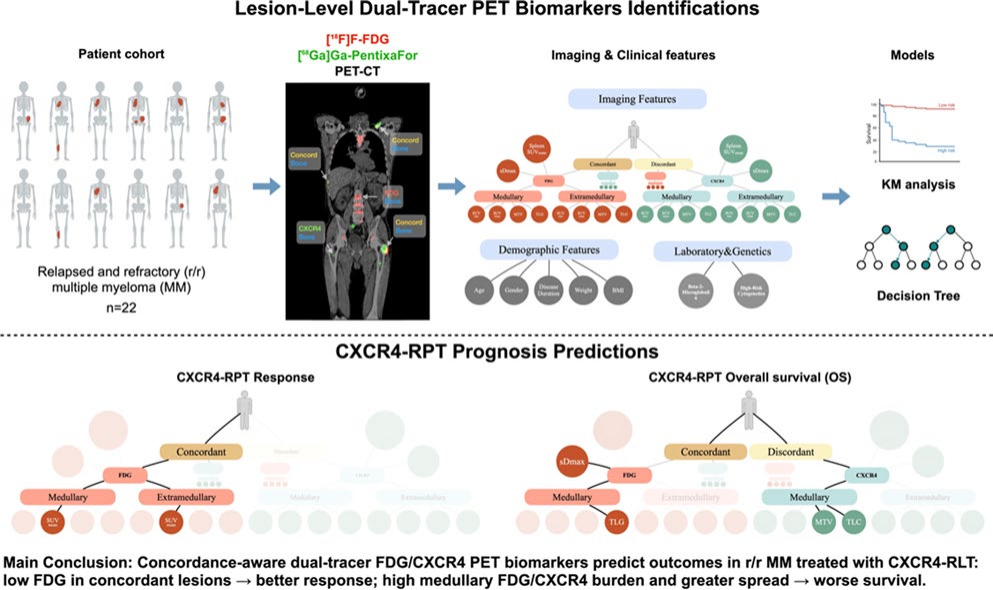

**Supplementary Information:**

The online version contains supplementary material available at 10.1007/s00259-026-07814-5.

## Introduction

In recent years, theranostic approaches in nuclear medicine, combining targeted positron emission tomography (PET) imaging and radiopharmaceutical therapy (RPT), were able to attract attention in a variety of malignant diseases due to their promising response rates and comparably beneficial side effect profile [1, 2]. However, to this date only theranostic concepts targeting solid tumors have successfully transitioned into routine clinical practice [3], including prostate-specific membrane antigen (PSMA)-directed theranostics in prostate cancer [4] and somatostatin receptor (SSTR)-directed approaches in neuroendocrine neoplasms [2]. Expanding modern theranostic concepts into hematologic neoplasms, C-X-C motif chemokine receptor 4 (CXCR4) has emerged as a promising target, as it is overexpressed on the tumor cell surface in a variety of hematologic neoplasms [5, 6]. Through the development of the PET-ligand [^68^Ga]Ga-PentixaFor [7] and its therapeutic equivalents [^177^Lu]Lu- and [^90^Y]Y-PentixaTher [8], CXCR4-directed theranostic options have become clinically available [9, 10].

First experiences with CXCR4-directed RPT yielded promising results in a variety of hematologic neoplasms [11–13], including advanced multiple myeloma (MM) [14]. However, treatment responses have been heterogeneous so far [12, 14], indicating the need for optimized patient selection. Current patient selection relies on qualitative assessment of CXCR4-expression (as determined by [^68^Ga]Ga-PentixaFor-PET) in vital MM lesions (as determined by [^18^F]FDG-PET). To date, no distinct predictive factors possibly enabling an optimized prediction of therapy response and thus better patient stratification before CXCR4-directed RPT have been identified. Furthermore, especially MM exhibits complex inter- and intratumoral heterogeneity [15], underlining the need for robust selection criteria before application of CXCR4-directed RPT.

Dual-tracer PET/CT offers a unique opportunity to assess both receptor expression (via [⁶⁸Ga]Ga-PentixaFor) and tumor metabolic activity (via [¹⁸F]FDG), providing complementary information about disease biology. In prostate cancer, dual-tracer PET/CT-imaging, applying a diagnostic PSMA-ligand and [^18^F]FDG before PSMA-directed RPT, could already demonstrate prognostic implications both on a patient- [16] as well as on a lesion-based level [17]. However, CXCR4-directed RPT protocols have yet to incorporate such quantitative, integrated imaging approaches in clinical decision-making. In current practice, patients with overt “mismatch” lesions (i.e., FDG^+^/CXCR4^−^) are generally excluded from therapy due to the concern of an insufficient treatment effect. While this binary exclusion may prevent ineffective interventions, it fails to leverage the full spectrum of quantitative imaging information—particularly in MM, where disease manifestations span a diverse array of biological phenotypes and anatomical sites [18]. A more refined, lesion-level analysis that accounts for both spatial distribution (e.g., bone vs. extramedullary) and dual-tracer uptake could offer a more precise basis for therapy guidance.

We hypothesized that quantitative imaging biomarkers extracted from dual-tracer PET/CT could potentially provide clinically actionable predictors of therapeutic response and survival following CXCR4-directed RPT. To test this hypothesis, we developed an automated pipeline for image registration, lesion segmentation, and feature extraction across both tracers. We conducted an exploratory, hypothesis-generating analysis in a retrospective cohort of 22 patients with relapsed and refractory (r/r) MM, applying interpretable machine learning models, including decision tree and survival tree analyses, to identify potential imaging-based predictors of treatment benefit. Our study takes a first step to move beyond simple positivity thresholds and proposes a quantitative, lesion-informed framework, aiming to optimize patient selection and outcome prediction in CXCR4-directed RPT that might also be translated to other theranostic concepts.

## Materials and methods

### Patient population

This retrospective study included 22 patients with r/r MM receiving 24 cycles of CXCR4-RPT who underwent dual-tracer PET/CT imaging prior to RPT at the University Hospital of Würzburg, Germany. All patients had histologically confirmed MM and had exhausted all standard therapy regimens. CXCR4-directed RPT was conducted on an individual basis according to the German Pharmaceutical Act (§ 13.2b). All patients signed written informed content prior to all procedures. The need for an approval for this analysis was waived by the local ethics committee due to the retrospective nature of this study (# 20220103 01).

### Imaging protocols

All patients underwent both [⁶⁸Ga]Ga-PentixaFor- and [¹⁸F]FDG-PET/CT scans within a 30-day interval (median 2 (1–27) days), to validate relevant CXCR4-expression in vital myeloma lesions before administration of CXCR4-directed RPT. 298 (189–340) MBq of [^18^F]FDG and 132 (43–165) MBq of [^68^Ga]Ga-PentixaFor were intravenously injected, respectively. Imaging was performed after 60 min using a Siemens Biograph mCT 64 or 128 system (Siemens Healthineers, Erlangen, Germany). PET scans were corrected for attenuation, scatter, and decay, and reconstructed using vendor-specified iterative algorithms. CT images were acquired for anatomical reference and attenuation correction. PET quantification was standardized using body-weight normalized SUV units.

### CXCR4-directed RPT and outcome assessment

CXCR4-directed RPT was administered followed by additional chemotherapeutic conditioning (CON) and subsequent autologous or allogeneic hematopoietic stem cell transplantation (HSCT), as described before [19]. In this cohort, in 3/24 (12.5%) cycles 7.8 (7.6–15.2) GBq of [^177^Lu]Lu-PentixaTher were administered and in 21/24 (87.5%) cycles 5.5 (2.4–7.4) GBq of [^90^Y]Y-PentixaTher were applied. In 2/24 (8.3%) cycles, patients additionally received subsequent radioimmunotherapy using Rhenium-188-labeled anti-CD66 antibodies (8.6 GBq or 12.7 GBq, respectively) before application of CON. Chemotherapeutic CON regimen to enforce myeloablation after CXCR4-RPT in this end-stage disease setting were selected on an individual-patient base by our institutions department of hemato-oncology, applying individualized dosages of Melphalan-, Treosulfan- or Fludarabine-based regimen. In 21/24 (87.5%) cycles, subsequent autologous HSCT was performed, 3/24 (12.5%) cycles were accompanied by allogeneic HSCT. Therapeutic response was assessed based on serum M-protein or free light-chain levels before RPT and approximately 6 weeks after completion of HSCT, if available. Response was defined as ≥ 50% reduction in serum M-protein or free light-chain levels, as adapted from the International Myeloma Working Group (IMWG) response criteria [20]. In 4/24 (16.7%) cycles, serum M-protein levels before/after RPT were not available for analysis, so an imaging-based response assessment was performed, if available. Therefore, we either analyzed [^18^F]FDG-PET/CT scans according to the IMPeTUs criteria [21] or whole body magnetic resonance imaging (MRI) according to the MY-RADS criteria [22]. Patients achieving complete response (CR), very good partial response (VGPR) and partial response (PR) were defined as responders, stable disease (SD) and progressive disease (PD) were defined as non-responders. Overall survival (OS) and progression free survival (PFS) were assessed using the day of CXCR4-RPT as day 0. Patients were stratified based on overall survival of ≥ 6 months after administration of CXCR4-directed RPT, a clinically relevant threshold in this end-stage disease setting.

### Imaging feature analysis

**Fig. 1:**
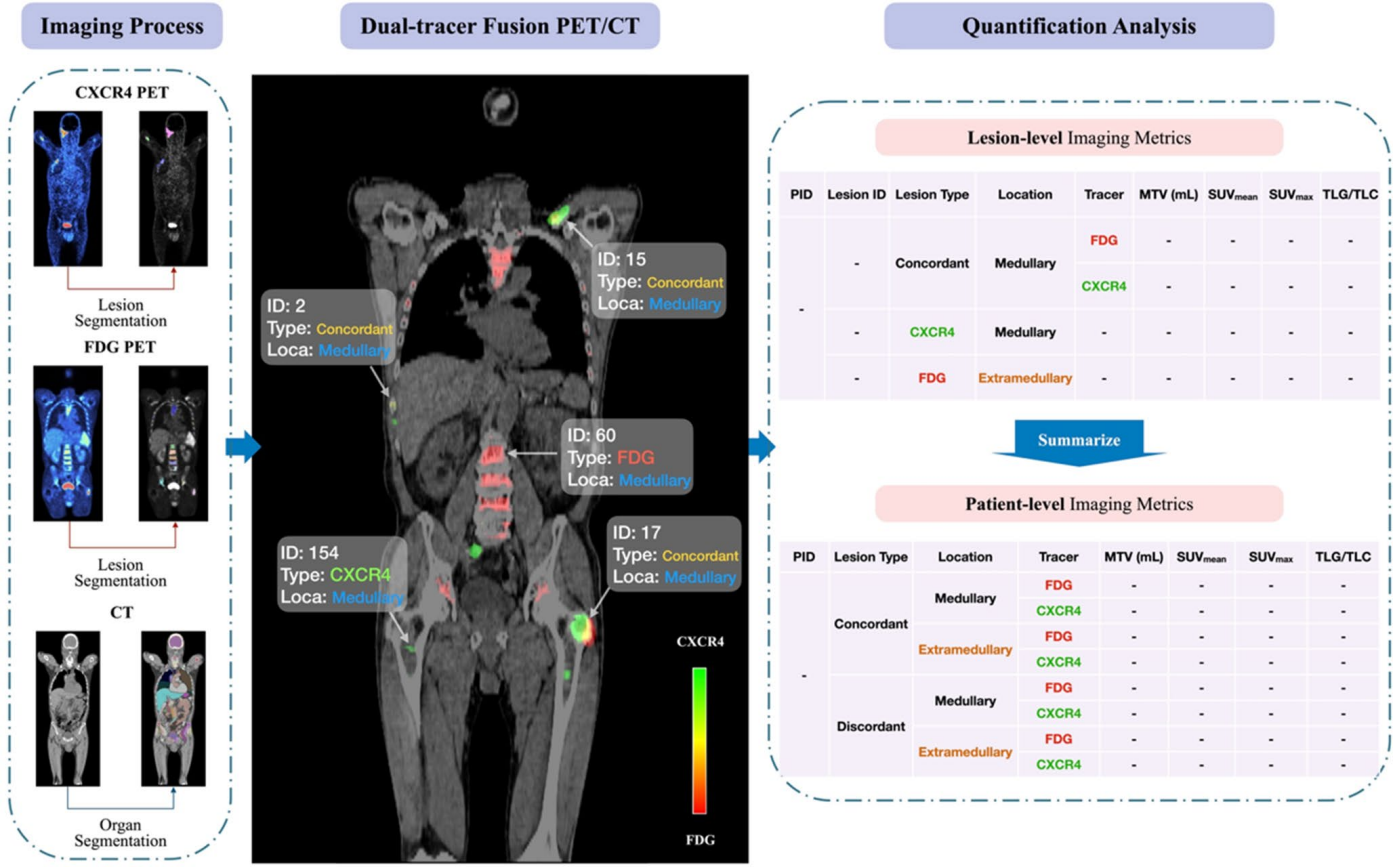
Workflow of the proposed CXCR4/FDG fusion imaging analysis. An end-to-end pipeline was developed for automated processing of [^68^Ga]Ga-PentixaFor- and [^18^F]FDG-PET/CT scans. The workflow included lesion segmentation and localization, dual-tracer image fusion, concordant lesion identification, and quantitative feature extraction at the lesion and patient levels. MTV, molecular tumor volume (in ml); TLC, total lesion CXCR4-expression; TLG, total lesion glycolysis

A fully automated image analysis pipeline was developed to enable lesion-level quantitative analysis across both tracers (Fig. 1). Myeloma lesion segmentation was first performed on [¹⁸F]FDG-PET using an nnU-Net-based model (Euclid software, Evomics Ltd.) [23]. The corresponding CT volumes from [¹⁸F]FDG- and [⁶⁸Ga]Ga-PentixaFor-PET scans were co-registered using rigid and deformable transformations, and the resulting transformation matrices were applied to align all PET volumes into a common anatomical space. Technical details are provided in the Supplementary Methods.

The initial [¹⁸F]FDG-PET segmentations were transferred to the [⁶⁸Ga]Ga-PentixaFor-PET images and subsequently refined by two experienced nuclear medicine physicians (ND, JSE) to ensure accurate delineation across both modalities. A lesion was defined as concordant if it was detected on both [¹⁸F]FDG- and [⁶⁸Ga]Ga-PentixaFor-PET, corresponding to overlapping lesion volumes exceeding 10% on the fused PET images. Lesion-level imaging features were then extracted, including SUV_mean_, SUV_max_, molecular tumor volume (MTV), total lesion glycolysis (TLG), and total lesion CXCR4-expression (TLC). Anatomical segmentation was performed on the co-registered CT images using TotalSegmentator [24]. Segmentation masks were propagated to PET space, and lesions were categorized as medullary or extramedullary based on ≥ 10% volume overlap with bone segmentations.

Lesion-level features were subsequently aggregated to the patient level. Bracket notation was used to specify tracer and lesion subset—for example, SUV_mean_[CXCR4 medullary concord] denotes the SUV_mean_ of [⁶⁸Ga]Ga-PentixaFor-PET over concordant medullary lesions, whereas TLG[FDG extramedullary discordant] denotes the [¹⁸F]FDG-PET-derived TLG of discordant extramedullary lesions.

The maximum standardized distance (sDmax) was computed for each tracer as the body surface–standardized distance between the two most distant lesions [25]. Finally, spleen SUV_mean_ was measured, excluding any splenic lesions when present [26].

### Clinical features

To account for possible impact of clinical parameters in our exploratory analysis of this heterogenous, end-stage cohort, we included cytogenetic characteristics of myeloma lesions for each patient and classified them as either high-risk-cytogenetics or non-high-risk-cytogenetics [27]. Furthermore, we included pre-RPT beta-2 microglobulin levels (in mg/l) as a possible prognostic factor of outcome. Additionally, individual personal features such as sex, age, body-mass-index (BMI) and disease duration were included into the analysis.

### Statistical analysis

Two primary endpoints were assessed: therapy response (responder vs. non-responder) and overall survival (OS). For therapy response, continuous variables, including imaging and clinical features, were compared between groups using Welch’s t-test, while binary variables such as sex and cytogenetic status were evaluated with Chi-square tests. A two-sided *p* < 0.05 was considered statistically significant. To further capture non-linear associations and interactions among predictors of response, we trained binary classification trees, with model performance estimated by repeated stratified 5-fold cross-validation (10 repeats). Feature attribution was quantified using SHAP (SHapley Additive exPlanations) and mean absolute SHAP values were aggregated across validation folds to obtain stable feature importance estimates. We report Hedges’ g (bias-corrected standardized mean difference) with 95% CIs for continuous features and odds ratios (ORs) with 95% CIs for binary features, alongside p values from Welch’s t-tests or Chi-square/Fisher’s exact tests.

For survival analysis, Kaplan–Meier (KM) curves were generated to visualize outcome stratification by all features, and group differences were assessed using the log-rank test. Hazard ratios (HRs) were approximated as event-rate ratios between groups. In addition, survival was dichotomized at 6 months (< 6 months vs. ≥6 months) to enable binary classification with decision trees, trained using the same cross-validation strategy and pruning procedures described above. SHAP values were again used to rank feature contributions, and SHAP-based rankings were employed to illustrate the relative impact of clinical and imaging features on survival outcomes. Given the exploratory, hypothesis-generating design and small sample, no multiple-comparison correction was applied. P-values are descriptive and interpreted alongside effect sizes.

## Results

### Patient population

24 cycles of CXCR4-directed RPT were conducted in 22 patients, as described above. In the two patients receiving an additional cycle of CXCR4-RPT, there was a timespan of 67 and 77 days in between the application of the respective cycles. Median age was 56 (42–71). 10/22 (45.5%) subjects were female. Patients had received median 5 (3–9) therapy lines before RPT, in 20/22 (90.9%) individuals, prior HSCT had been conducted. CXCR4-directed RPT was performed median 49.5 (10–198) months after first diagnosis. High-risk cytogenetics were present in 14/24 (58.3%) cycles and beta-2 microglobulin levels before RPT were median 2.95 (1.4–13.8) mg/l. Further patient characteristics are depicted in Table 1.

**Table 1:** Patient characteristics

Age (y)	56 (42–71)
Sex (*n*)	
Male	12/22 (54.5%)
Female	10/22 (45.5%)
BMI (kg/m^2^)	26 (18–43)
Subtype of Multiple myeloma (n)	
* IgG-Lambda*	5/22 (22.7%)
* IgG-Kappa*	4/22 (18.2%)
* IgA-Lambda*	4/22 (18.2%)
* Kappa light-chain*	4/22 (18.2%)
* IgM-Kappa*	2/22 (9.1%)
* IgA-Kappa*	2/22 (9.1%)
Asecretory	1/22 (4.5%)
Prior treatment lines (n)	5 (3–9)
Prior HSCT (n)	22/24 (91.7%)
HSCT (n)	
* Autologous*	21/24 (87.5%)
* Allogeneic*	3/24 (12.5%)

Median and range (min – max) are displayed. *BMI* Body-mass-index, *HSCT* Hematopoietic stem cell transplant

### Dual-Tracer imaging before therapy

**Fig. 2:**
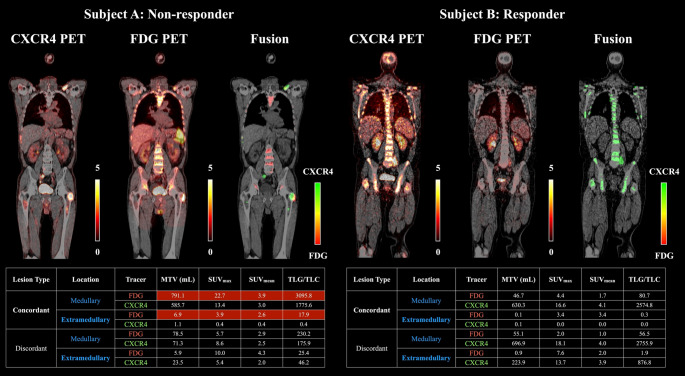
Representative patients illustrating tracer concordance and lesion phenotypes. Coronal PET/CT images are shown for a non-responder (Subject A) and a responder (Subject B): [^68^Ga]Ga-PentixaFor-PET, [^18^F]FDG-PET, and fused overlays. In the fusion, discordant CXCR4-positive lesions are rendered green and FDG-positive red; concordant lesions appear yellow (defined as > 10% volumetric overlap between tracers). The accompanying tables summarize lesion metrics by location (medullary vs. extramedullary), concordance (concordant vs. discordant), and uptake parameters, including MTV, SUV_max_, SUV_mean_, and TLG/TLC. Subject A shows diffuse, predominantly concordant disease with comparatively higher uptake parameters in [^18^F]FDG-PET —reflected by elevated SUV_mean_ in extramedullary- and medullary-concordant lesions. In contrast, Subject B presents a substantial lesion burden across medullary and extramedullary sites, but lesions are mostly CXCR4-only (discordant) with lower uptake in [^18^F]FDG-PET. These exemplars are consistent with the cohort-level findings. MTV, molecular tumor volume (in ml); TLC, total lesion CXCR4-expression; TLG, total lesion glycolysis

Pre-treatment dual-tracer PET/CT with [¹⁸F]FDG and [⁶⁸Ga]Ga-PentixaFor identified 1,352 lesions across all patients (median 64.5 per patient; range, 20–109). Of these, 661 were CXCR4-only (discordant) lesions (medullary, *n* = 420, extramedullary, *n* = 241), 171 were FDG-only (discordant) MM manifestations (medullary, *n* = 60, extramedullary, *n* = 111), and 520 were concordant on both tracers. Most patients exhibited widespread concordant disease, with a median of 40.0% (range, 5.6–87.1%) concordant lesions per patient. Extramedullary involvement was present in all subjects, comprising a median 39.8% (range, 4.8–77.5%) of lesions per patient. Figure 2 illustrates typical phenotypes: A patient not responding to CXCR4-directed RPT with diffuse, predominantly concordant (yellow) disease and higher [¹⁸F]FDG-uptake in both medullary and extramedullary compartments, and a patient rated as a responding to RPT with substantial lesion burden that is largely CXCR4-only (green, discordant) with comparatively lower [¹⁸F]FDG-uptake. These exemplars mirror the cohort-level associations described below.

### Response assessment

Of the 24 administered treatment cycles, serological response data was available in 20 cycles; among these, 10/20 (50%) achieved an objective response to CXCR4-directed RPT [VGPR 3/10 (30%); PR in 7/10 (70%)]. The remaining 10/20 cycles (50%) exhibited stable [7/10, (70%)] or progressive [3/10 (30%)] disease. In 2/24 (8%), imaging-based response assessment was performed (one CR and PD, each). The remaining 2/24 (8%) lacked sufficient follow-up for definitive categorization and were considered non-responders in the primary analysis. Median OS for the cohort was 198 days (56–665). Median OS was 209 days (94–665) among responders and 192 days (56–503) among non-responders.

### Key predictive imaging features for therapy response

**Fig. 3:**
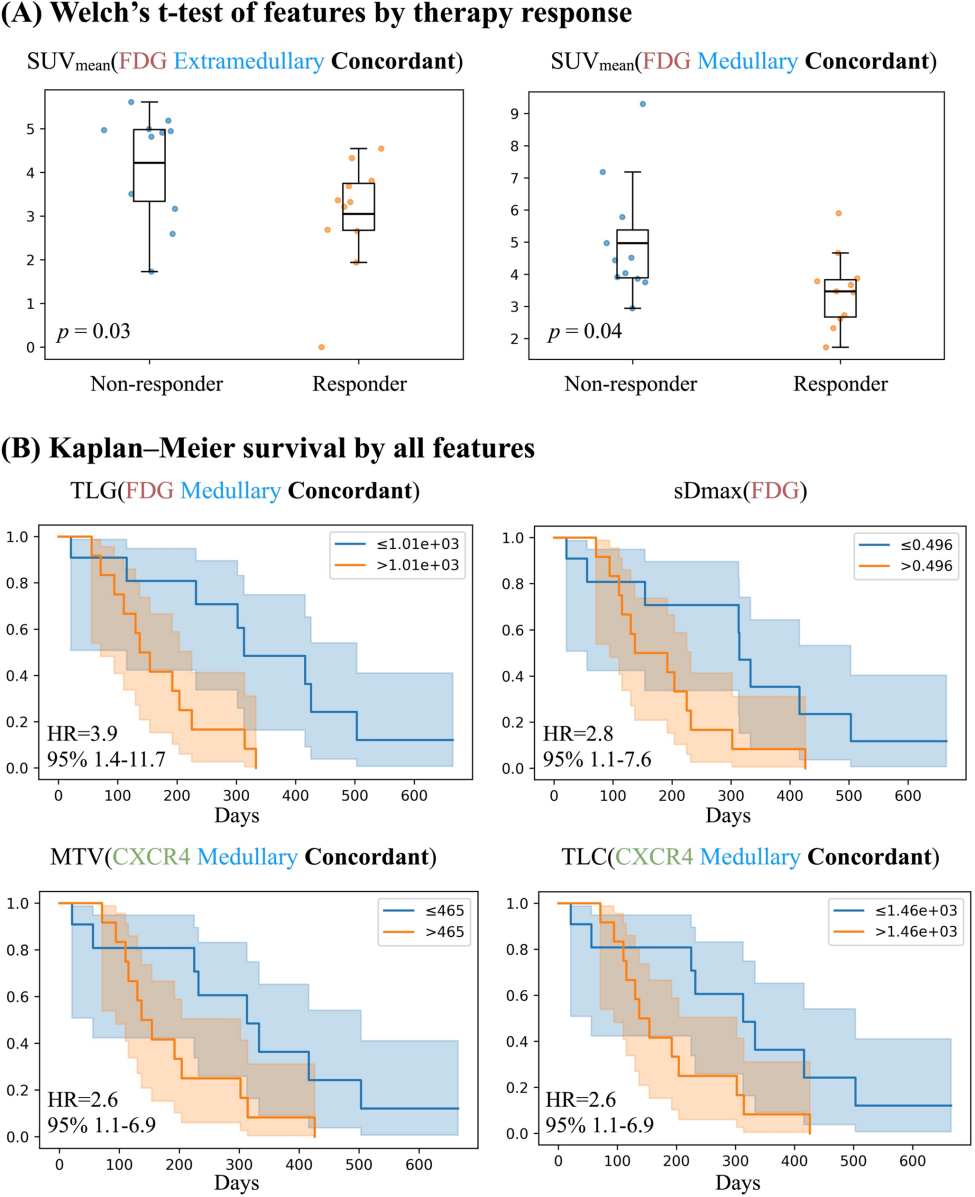
Univariate analysis of response and overall survival (OS). (**A**) Welch’s t-tests comparing responders vs. non-responders across all features; SUV_mean_[FDG extramedullary concord] and SUV_mean_[FDG medullary concord] showed significant differences between groups (*p* < 0.05). Boxplots display the interquartile range (box), median (line), and range (whiskers) as well as individual data points; p values from two-sided Welch’s t-tests. (**B**) Kaplan–Meier curves for overall survival stratified by four features dichotomized at the cohort median: TLG[FDG medullary concordant], sDmax[FDG], MTV[CXCR4 medullary concordant], and TLC[CXCR4 medullary concordant]. Shaded bands indicate 95% CIs. HRs are event-rate ratios; p values from log-rank tests. MTV, molecular tumor volume (in ml); sDmax, maximum standardized inter-lesion distance; TLC, total lesion CXCR4-expression; TLG, total lesion glycolysis

**Fig. 4:**
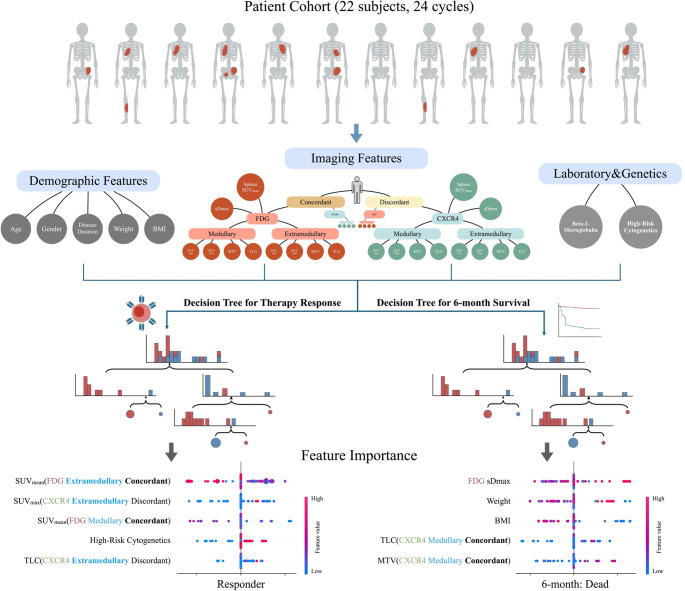
Decision tree–based stratification and SHAP-derived feature importance for predicting CXCR4-RPT response and 6-month survival. Feature construction included demographics, imaging features (FDG- and CXCR4-PET/CT; medullary vs. extramedullary; concordant vs. discordant), and laboratory/genetic variables. Two parallel binary decision-tree models were trained: (i) therapy response (responder vs. non-responder) and (ii) 6-month survival (< 6 vs. ≥ 6 months). SHAP summary plots illustrate the relative importance and direction of effect of the top predictors. MTV, molecular tumor volume; TLG, total lesion glycolysis; TLC, total lesion CXCR4-expression; sDmax, maximum standardized inter-lesion distance; SHAP, SHapley Additive exPlanations

Welch’s t-tests identified SUV_mean_[FDG extramedullary concordant] and SUV_mean_[FDG medullary concordant] as significantly lower in responders than non-responders, with mean-difference 95% CIs of − 1.8 to − 0.06 (*p* = 0.03) and − 1.8 to − 0.01 (*p* = 0.04), respectively (Fig. 3A). In the decision tree analysis, both features ranked among the top three predictors of response by SHAP. Additional influential variables selected by the decision tree included SUV_max_[CXCR4 extramedullary non-concordant], high-risk cytogenetics, and TLC[CXCR4 extramedullary non-concordant] (Fig. 4).

SHAP analyses indicated that higher values of SUV_mean_[FDG extramedullary concordant] and SUV_mean_[FDG medullary concordant] consistently shifted predictions toward non-response, whereas higher SUV_max_[CXCR4 extramedullary non-concordant] and TLC[CXCR4 extramedullary non-concordant] shifted predictions toward response. Interestingly, high-risk cytogenetics contributed additively toward better response in this end-stage disease setting. Directionality was stable across cross-validation folds (≥ 80% inter-fold sign agreement).

### Key predictive imaging features for 6-month survival

KM analyses showed significantly worse overall survival for patients with higher TLG[FDG medullary concordant] (HR ≈ 3.9; 95% CI 1.4–11.7), greater sDmax[FDG] (HR ≈ 2.8; 95% CI 1.1–7.6), higher MTV[CXCR4 medullary concordant] (HR ≈ 2.6; 95% CI 1.1–6.9), and higher TLC[CXCR4 medullary concordant] (HR ≈ 2.6; 95% CI 1.1–6.9); all log-rank *p* < 0.05 (Fig. 3B). In the 6-month survival decision tree model, these variables again ranked among the top 5 SHAP features, alongside weight and BMI (Fig. 4).

Consistent with the KM results, SHAP directionality showed that higher TLG[FDG medullary concordant], sDmax[FDG], MTV[CXCR4 medullary concordant], and TLC[CXCR4 medullary concordant] shifted predictions toward early mortality (< 6 months). In contrast, higher BMI was associated with a lower predicted risk of early mortality. The direction for weight was retained in the ranking but exhibited lower inter-fold stability.

## Discussion

This study finds that quantitative imaging features derived from dual-tracer PET/CT—specifically those extracted from concordant lesions— could possibly play a role in enhancing prediction of both response and survival following CXCR4-directed RPT in patients with r/r MM. By integrating [¹⁸F]FDG- and [⁶⁸Ga]Ga-PentixaFor-PET into a unified lesion-level analysis framework, our study suggests biologically and clinically distinct imaging signatures that could support outcome prediction beyond current qualitative assessment standards. Importantly, the univariate analyses and decision tree models converged on a key finding: the most predictive biomarkers in our exploratory analysis originated primarily from concordant lesions, i.e., lesions with increased CXCR4-expression and glycolytic activity. These lesions likely represent viable, metabolically active disease that influences response to CXCR4-directed RPT. Our results suggest that lower metabolic activity as within concordant lesions as determined by [¹⁸F]FDG-PET is associated with better response. This pattern supports the concept that higher uptake in [¹⁸F]FDG-PET might represent a more “aggressive” myeloma subtype and is therefore less likely to respond to therapy.

Our data indicate partial convergence rather than complete separation of response and survival predictors. Both endpoints share a core FDG–medullary–concordant signal: higher SUV_mean_[FDG medullary concordant] and higher TLG[FDG medullary concordant]— reflecting the intensity and volumetric burden of a possibly more “aggressive” myeloma phenotype at the time of RPT—were unfavorable, associated with non-response and shorter OS. Divergence emerges in secondary determinants. Survival was additionally driven by CXCR4-expressing myeloma burden in the marrow (higher MTV and TLC) and by spatial dispersion (sDmax), highlighting the importance of overall disease burden in this end-stage setting. In other lymphoma entities, such as diffuse large B-cell lymphoma, the sDmax is hereby already an established parameter to quantify disease spread and holds prognostic value independent from other disease burden parameters including MTV [28]. In MM however, the sDmax is currently not routinely determined with only scarce data available. A recent analysis in newly diagnosed MM patients demonstrated its independent predictive value for event-free survival [29], which is in line with the results of our analysis in an advanced-stage disease setting. BMI showed a modest protective association, which could be linked to a higher overall fitness of the subjects and therefore an improved overall survival. In contrast, response modeling assigned added body weight to extramedullary CXCR4-dominant, discordant metrics (e.g., SUVmax/TLC). Collectively, these patterns suggest that the prevalent glycolytic medullary myeloma burden sets a common baseline risk for both endpoints, whereas extent of CXCR4-positive disease and host factors (e.g., BMI) might modulate long-term survival more than initial treatment sensitivity.

In addition to evaluating patients` suitability for CXCR4-directed RPT and performing response/survival prediction by deriving quantitative PET parameters as suggested in the current analysis, dual-tracer imaging using [¹⁸F]FDG- and [⁶⁸Ga]Ga-PentixaFor-PET might hold further capabilities improving diagnostic and therapeutic procedures in hematologic neoplasms as CXCR4-directed PET/CT might demonstrate superior lesion detection rates [30]. However, previous studies revealed a more heterogenous CXCR4 expression in advanced-stage MM [31–33], underlining the need for further research to define the optimal time points for both imaging and therapy, also taking into account the influence of external factors such as applied therapy regimen or concomitant medication on chemokine receptor expression.

With regard to other emerging therapeutic approaches in the r/r MM setting, such as CAR-T-cell therapy, prognostic and predictive models based on pre-therapy [^18^F]FDG have been proposed in patients with B-cell lymphoma, demonstrating effective prediction of not only PFS and OS, but also side-effects of therapy, especially when integrating further clinical variables [34]. For MM, higher MTV determined by [^18^F]FDG-PET was also shown to be associated with worse prognosis [35]. Based on these findings, one might imagine not only improved diagnostic performance and improved predictive value by combining the two PET-agents, but maybe also valuable predictive information on side-effects of therapy. This could be particularly the case for potential immunogenic side effects, considering that both [^18^F]FDG- and [^68^Ga]Ga-PentixaFor-PET hold capabilities in visualizing inflammatory processes. Therefore, PET-features deducted from dual-tracer imaging could possibly reveal enhanced predictive potential not only for response and prognosis in CXCR4-directed RPT, but also in the context of other therapeutic approaches, maybe also expanding the predictive potential to side-effects of therapy.

The current initial results were made possible by our fully automated image analysis pipeline, which facilitated precise lesion segmentation, dual-tracer image fusion, and feature extraction at the lesion, organ, and patient levels. The pipeline’s reproducibility, coupled with model explanations via SHAP, provides a transparent, scalable foundation for a possible theranostic integration and prospective decision support in MM. Nonetheless, several limitations must be acknowledged. The study’s retrospective nature and modest cohort size limit its generalizability. Although repeated stratified cross-validation and aggregation of SHAP values across folds were applied to mitigate model instability, feature importance estimates should be interpreted with caution. Given the limited number of observations relative to model complexity, the observed patterns of feature attribution are considered exploratory and may not reflect stable or generalizable associations. These results should therefore be viewed as hypothesis-generating rather than confirmatory. Consequently, the decision-tree models and corresponding SHAP interpretations are not intended for predictive application at the individual-patient level, but rather to provide insight into potential nonlinear relationships warranting further validation. Additionally, scans were acquired using two different PET-scanners without cross-scanner harmonization in the present study, possibly limiting the comparability of SUV-based metrics and volumetric parameters by differences in spatial resolution, reconstruction algorithms and image noise characteristics. Furthermore, the dynamic disease progression in MM might further limit the comparability of the respective CXCR4- and FDG-PET scans, even when performed in a short time span. Our results might additionally be confounded by using the FDG-PET based segmentation as a primary lesion definition, possibly hampering the detection of FDG-only or CXCR4-only lesions. The interpretability of our results is further limited by the heterogeneity of the cohort in this end-stage disease setting, in which patients had already received all established treatment options and, in some cases, additional experimental therapies. In addition, two patients received additional radioimmunotherapy, and in treatment cycles in which serological response assessment was not feasible, imaging-based response assessment was conducted. Moreover, due to the retrospective setting, only few clinical parameters and established risk factors could be included into the analysis. Future prospective studies with larger and more diverse populations, standardized time points of imaging and application of RPT as well as longitudinal follow-up will be essential to confirm and refine these imaging biomarkers for clinical use.

Regarding the need to perform HSCT after conducting CXCR4-RPT due to its myeloablative effects, recent results as presented in the GMMG ReLApsE study might suggest the critical re-evaluation of a therapeutic regimen including salvage HSCT in an r/r MM setting, as HSCT alone could not demonstrate favorable outcome when compared to standard maintenance therapy [36]. However, there is no clear comparability to the setting presented in this manuscript, as the addition of CXCR4-RPT could not only be expected to have independent anti-myeloma effects, but also, due to a deeper myeloablation, possible synergistic effects with the HSCT regimen. Nevertheless, it should be taken into consideration for future applications of our presented approach that potential additive or synergistic effects between CXCR4-RPT and HSCT might be less pronounced in an r/r MM setting.

## Conclusion

In this exploratory analysis in a retrospective cohort of r/r MM treated with CXCR4-directed RPT, quantitative dual-tracer PET/CT features were associated with both early treatment response and overall survival. High [¹⁸F]FDG-uptake in medullary concordant lesions aligned with non-response and shorter survival. CXCR4-PET derived features and sDmax added further survival information. By proving the feasibility of our deep-learning based dual tracer PET analysis, deriving features from an automated, lesion-level, concordance-aware pipeline, our findings suggest that integrating [¹⁸F]FDG- and [^68^Ga]Ga-PentixaFor-PET information at lesion level might capture complementary aspects of disease biology and in the future may help refine patient selection and counseling for CXCR4-directed RPT. Although needing validation in larger and possibly prospective cohorts to possibly influence clinical decision making, our methodology might be applicable to other dual-tracer approaches.

## Supplementary Information

Below is the link to the electronic supplementary material.

Supplementary Material 1

## Data Availability

Data are available for legitimate researchers who request it from the corresponding author.
