# Supplementary material for: Lesion-level dual-tracer PET biomarkers predict prognosis in multiple myeloma treated with CXCR4-directed radiopharmaceutical therapy
Source: Eur J Nucl Med Mol Imaging. 2026 Feb 18;53(7):4560–71. doi: 10.1007/s00259-026-07814-5 (PMC13197444; doi:10.1007/s00259-026-07814-5)
Supplement: Supplementary file 1 — Supplementary Material 1 [file 259_2026_7814_MOESM1_ESM.docx]

# Supplementary materials

**MATERIALS AND METHODS**

**Imaging Feature Analysis**

A fully automated, end-to-end pipeline was developed for lesion-level quantitative analysis across both tracers. Lesion segmentation on [¹⁸F]FDG-PET was performed using an institutional nnU-Net–based model, which automatically configures a 3D U-Net–style architecture based on dataset fingerprinting. Model training combined the AutoPET challenge dataset with a large, institutionally curated PET/CT cohort approved by the local ethics committee. To prevent data leakage, the study cohort used for analysis was strictly independent of all training data. All inferences were performed using a single frozen checkpoint. PET volumes were processed in the native patient space defined by the [¹⁸F]FDG-CT, employing nnU-Net’s default tiling strategy and test-time augmentation. The resulting FDG lesion label maps were retained in this reference space for subsequent analysis.

Cross-tracer registration was performed by aligning the [⁶⁸Ga]Ga-PentixaFor CT to the FDG-CT through a rigid six-degree-of-freedom transformation followed by a multi-resolution B-spline deformable registration. The composite transformation was applied to the corresponding PET images using tri-linear interpolation for intensity data and nearest-neighbor interpolation for segmentation labels, thereby placing both tracers in a shared anatomical coordinate system. Registration quality was visually confirmed by evaluating osseous landmarks; cases with residual misalignment exceeding approximately 2 mm were re-registered prior to analysis.

Lesion masks from FDG- and PentixaFor-PET were subsequently reviewed in a blinded fashion by two experienced nuclear medicine physicians. Minor manual refinements were performed where necessary to ensure accurate delineation across both tracers. A lesion was defined as concordant if it was visible on both tracers and demonstrated at least 10% volumetric overlap within the co-registered space. Lesions not fulfilling this criterion on one tracer were classified as discordant for that tracer. Lesions smaller than the minimum resolvable volume (three voxels) or located outside the CT body mask were excluded to avoid spurious measurements. The concordance definition was independently verified by two physicians.

Anatomical context was derived from the co-registered CT using TotalSegmentator to generate comprehensive whole-body organ masks. These were propagated to PET space using the same transformation fields, and each lesion was classified as medullary when ≥10% of its volume overlapped with the skeletal system, or extramedullary otherwise.

Quantitative PET metrics were computed in physical standardized uptake value (SUV) units derived from DICOM headers, with injected activity and body weight corrected for radioactive decay. For each finalized lesion on each tracer, we extracted SUV_max_, SUV_mean_, and metabolic (or molecular) tumor volume (MTV). Total lesion glycolysis (TLG) was defined as MTV[FDG] × SUV_mean_[FDG], and total lesion CXCR4 uptake (TLC) as MTV[CXCR4] × SUV_mean_[CXCR4]. Patient-level summaries were generated by aggregating lesion-level features according to tracer concordance and anatomical category.
